# Bifurcation analysis of a tuberculosis progression model for drug target identification

**DOI:** 10.1038/s41598-023-44569-7

**Published:** 2023-10-16

**Authors:** Eliezer Flores-Garza, Rogelio Hernández-Pando, Ibrahim García-Zárate, Pablo Aguirre, Elisa Domínguez-Hüttinger

**Affiliations:** 1 Departamento de Biología Molecular y Biotecnología, Instituto de Investigaciones Biomédicas, Universidad Nacional Autónoma de México, Ciudad Universitaria, 04510 Mexico, Mexico; 2 Sección de Patología Experimental, Departamento de Patología, Instituto Nacional de Ciencias Médicas y Nutrición Salvador Zubirán, Vasco de Quiroga 15, Belisario Domínguez Secc. 16, Tlalpan, 14080 Mexico City, Mexico; 3 Facultad de Ciencias, Universidad Nacional Autónoma de México, Ciudad Universitaria, Coyoacán, 04510 Mexico City, Mexico; 4 Departamento de Matemática, Universidad Técnica Federico Santa María, Casilla 110-V, Valparaíso, Chile

**Keywords:** Differential equations, Dynamical systems, Multistability, Regulatory networks, Tuberculosis

## Abstract

Tuberculosis (TB) is a major cause of morbidity and mortality worldwide. The emergence and rapid spread of drug-resistant *M. tuberculosis* strains urge us to develop novel treatments. Experimental trials are constrained by laboratory capacity, insufficient funds, low number of laboratory animals and obsolete technology. Systems-level approaches to quantitatively study TB can overcome these limitations. Previously, we proposed a mathematical model describing the key regulatory mechanisms underlying the pathological progression of TB. Here, we systematically explore the effect of parameter variations on disease outcome. We find five bifurcation parameters that steer the clinical outcome of TB: number of bacteria phagocytosed per macrophage, macrophages death, macrophage killing by bacteria, macrophage recruitment, and phagocytosis of bacteria. The corresponding bifurcation diagrams show all-or-nothing dose–response curves with parameter regions mapping onto bacterial clearance, persistent infection, or history-dependent clearance or infection. Importantly, the pathogenic stage strongly affects the sensitivity of the host to these parameter variations. We identify parameter values corresponding to a latent-infection model of TB, where disease progression occurs significantly slower than in progressive TB. Two-dimensional bifurcation analyses uncovered synergistic parameter pairs that could act as efficient compound therapeutic approaches. Through bifurcation analysis, we reveal how modulation of specific regulatory mechanisms could steer the clinical outcome of TB.

## Introduction

Tuberculosis (TB) is an infectious disease caused by the bacillus *Mycobacterium tuberculosis* (Mtb)^1^. It is considered the oldest pandemic and according to the World Health Organization (WHO) in 2021 there were 1.6 million deaths due to TB and 10.6 million people suffered active disease^2^, tuberculosis being the leading cause of death from a single infectious agent before the SARS-CoV-2 pandemic. In endemic countries, the initial or primary TB infection establishes itself in the lungs during childhood and is usually controlled by the immune system; only 10% of these cases will suffer progressive disease. In this primary TB infection, even in those cases that are controlled by the immune system, not all bacteria are eliminated; some bacteria remain in the tissues in a quiescent state with little or no reproductive activity for the rest of the life of the infected individual. This infectious state is called latent infection, it is clinically asymptomatic and a quarter of humanity suffers from this condition but only 10% of this population will develop active TB at some point in their lives^3^. TB is treated with a combination of 4 antibiotics for 6 to 9 months, which results in a high rate of treatment abandonment. This situation has provoked relapses and the emergence of Multidrug-resistant TB (MDR-TB)^4, 5^, which further complicates treatment by increasing its cost and toxicity^5^.

The emergence and rapid spread of drug-resistant^4^ and hyper virulent^6^. *M. tuberculosis* strains have created the necessity to develop novel treatments that are more effective and less toxic. Present-day conditions in tuberculosis in vivo experimental assays are characterized by restricted lab capacity, insufficient funds, low number of laboratory animals due to efforts of Animal Ethics Committees and outdated technology. A systems-level approach that quantitatively studies this progressive disease and overcomes current wet lab limitations is much needed. Systems biology is a discipline capable of proposing and validating hypotheses regarding complex human diseases that currently prevail^7–9^. Through mechanistic modelling, TB has been studied across multiple life scales and mathematical formalisms^10^, from host–pathogen interactions at the cellular level^11^ to single hybrid multi-compartment models of granuloma formation^12^. Thus, researchers have been able to describe some physiological determinants behind the persistence of a mycobacterial infection^13^ and predict how certain cellular interactions lead to different disease outcomes^14^.

Our previously proposed tuberculosis progression model^15^ is a tool that allows creating and testing hypotheses regarding disease outcome. The mathematical model describes the key regulatory mechanisms that account for the pathological progression of TB through three phases observed on in vivo mouse models. Previously^15^, we showed how random variations in model parameters that quantify the strength of the individual reactions of our mechanistic model segregate the long-term behavior into two qualitatively different phenotypes: bacterial clearance or persistent infection. Therefore, we speculated that specific model parameters could act as knobs that can steer the model to a desired outcome. To address this, a more comprehensive analysis on the influence of parametric variations on model variables dynamics is needed. Bifurcation analysis is a tool to map parameter variations to the outcome of a dynamical system^16, 17^. Several types of bifurcations can be predicted by Early Warning Signals^18, 19^ that could serve as risk biomarkers to stratify patients before they develop symptoms.

Here we systematically explore the effect of parameter variations on disease outcomes with the objective of identifying and characterizing the key mechanisms that determine the clinical phenotype. Our results pinpoint the five bifurcation parameters that segregate the phenotypic space into all-or-nothing long-term behaviors. We further explore the impact of the disease stage on the resulting bifurcation diagrams to identify the precise window of time in which treatment would be more effective. We complemented this analysis by investigations on the effects of parameter variations on the pace of progression across disease stages and uncovered additional constraints for optimal treatment scheduling. Moreover, this analysis suggested that latent TB can be reproduced by our model—originally developed for progressive TB—by adequately changing parameters to mimic genotypes with a significantly slower progression between disease stages. Finally, simultaneous variations of bifurcation parameter pairs identified synergistic effects that could inform the design and optimization of compound treatments.

Through bifurcation analysis, our model proves to be a powerful tool to quantitatively assess the influence of its mechanisms in determining the dynamic phenotype, opening the door to more in silico experimentation that will enable the design of optimal treatments that consider the right doses for the right patient at the right time.

## Results

### Mathematical model of the immunopathological progression of TB

**Figure 1:**
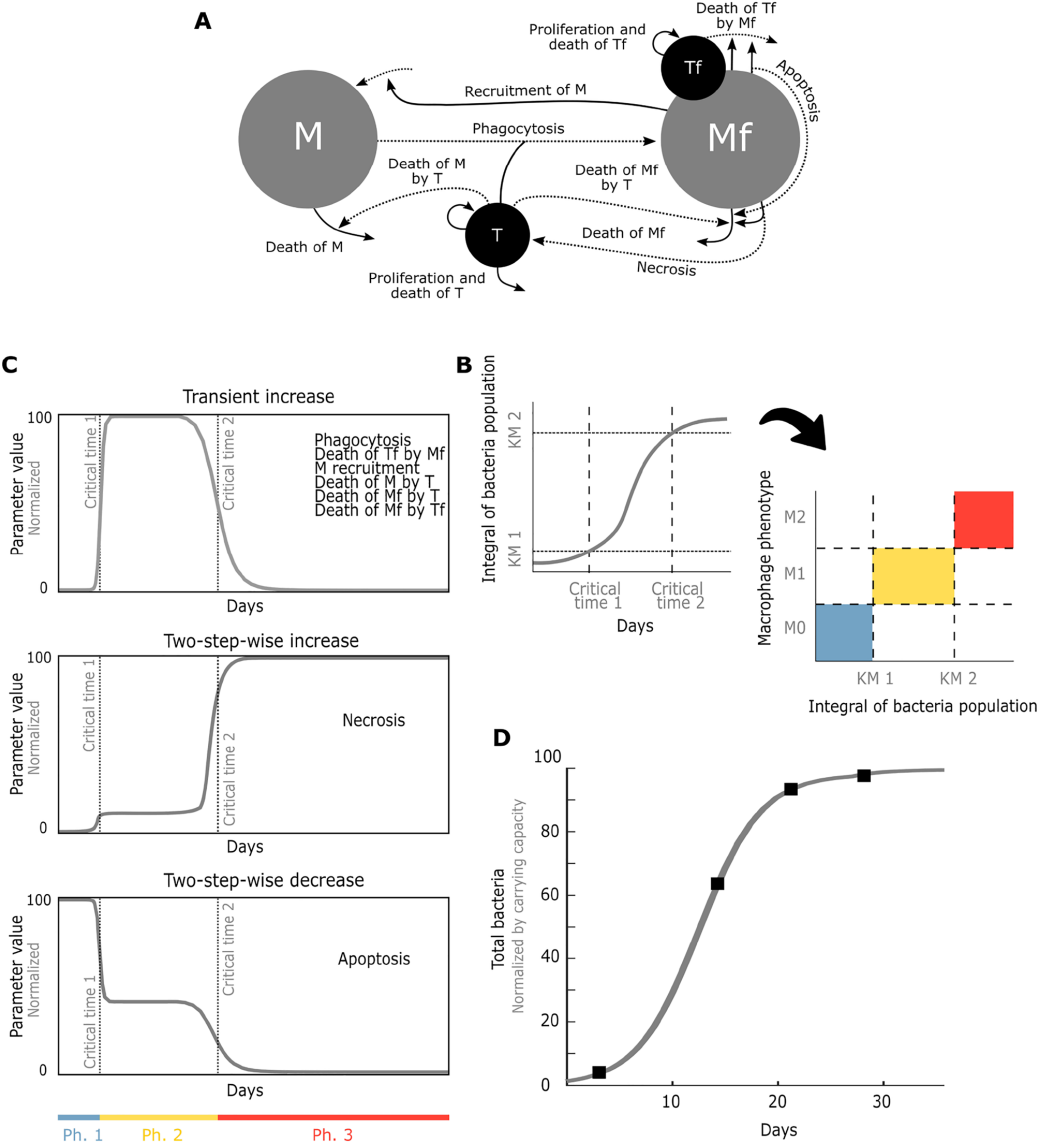
Mathematical model of the immunopathological progression of TB. (**A**) Graphical representation of the key mechanisms that describe the regulatory interplay between macrophages and bacteria. Dotted lines represent phase-dependent rates. (**B**) Historic bacteria population triggers phase changes when the threshold values *KM1* and *KM2* are surpassed at critical times 1 and 2. (**C**) Phase changes are reflected by adaptations in the values of the model parameters described in Table 2, which can transiently increase (top), stepwise increase (middle) or stepwise decrease (bottom)*.* (**D**) The mathematical model with nominal parameters taken from^17^ reproduces the validation data set extracted from^48^, corresponding to BALBc mice challenged intratracheally with 2.5 × 10^5^ CFU of H37Rv bacteria.

We previously^15^ proposed and calibrated a mathematical model of TB that captures the course of progressive TB in mice. The model is a mechanistic representation of the regulatory interplay between macrophages, which are the key immunological players in the host response to TB infection, and the bacteria *M. tuberculosis*. We distinguish between free and phagocytic forms of bacteria and macrophages with the four state variables: free macrophages ($M$*),* macrophages containing phagocyted *M. tuberculosis* ($Mf$), free *M. tuberculosis* ($T$) and phagocyted *M. tuberculosis* ($Tf$), which interact dynamically through a series of mechanisms depicted in Fig. 1A and described in the “Methods” section. A key feature of our model is that it explicitly represents progression through three experimentally observed phases: Phase 1 (Ph1), a preparation phase where non activated (M0) macrophages interact with the bacteria^20^; Phase 2 (Ph2), a pro-inflammatory phase where adaptive immune response activates the M1 macrophages^21^, and Phase 3 (Ph3), an anti-inflammatory phase where macrophages acquire a M2 phenotype^22^. Bacteria react to the phenotypic changes of macrophages by adapting their virulence through these 3 phases^23–25^. Our simple model reflects these complex processes by allowing specific parameters to dynamically adapt to persisting infection. Namely, parameters associated with macrophage pathogen-response capabilities (phagocytosis, cell recruitment, antimicrobial mechanisms) and those associated with bacterial virulence (killing rate of macrophages by bacteria); resulting in three phase-specific parameter sets (Tables 1 and 2). The transition between phases is modelled by inputting the time integral of *M. tuberculosis* to switch-like value changes in modulable parameters (Fig. 1B), as described in the “Methods” section. This integral-over-time of *M. tuberculosis* represents the history of infection in the respiratory tract, enacted by slowly activating and decaying Dendritic Cells that have migrated from the site of infection to the lymph nodes^26^. In turn, switch-like changes in modulable parameter values (Table 2) reflect phenotypic adaptations of *M. tuberculosis*^27^ and macrophages^28^ to abruptly changing inflammatory microenvironments occurring through T-cell differentiation triggered by sufficiently large accumulation of Dendritic Cells^29^ (Fig. 1C). This way, the historic population of bacteria functions as a capacitor triggering phase changes when surpassing the threshold values. This occurs at *critical time 1*, the time at which the change from Ph1 to Ph2 occurs, and *critical time 2*, when Ph2 changes to Ph3. Model Eq. (1) are described in the “Methods” section. The parameter values in Tables 1 and 2 were obtained previously^15^ by parameter optimizations using multiple in vivo and in vitro datasets. Here we show how the model reproduces a validation dataset derived from a progressive pulmonary TB mouse model assay that was not used for the model calibration (Fig. 1D).

**Table 1:** Model parameters.

Description	Parameter	Nominal value (Ph1) (adapted from^17^)	LTBI (Ph1) (heuristically determined)	Units
Death of *M*	${\upbeta }_{1}$	$0.002$	0.003	1/d
Proliferation of *T*	${\mathrm{\alpha }}_{2}$	$2.67$	0.07	1/d
Proliferation of *Tf*	${\mathrm{\alpha }}_{3}$	$1.47$	0.81	1/d
# T phagocytized by *M*	$\mathrm{PTf}$	19.2	3.1	$\frac{\mathrm{CFU}}{\mathrm{Mac}}$
Carrying capacity	$\mathrm{K}$, $\widetilde{\mathrm{K}}$	$2.4\times {10}^{7}$	6.5 $\times {10}^{7}$	$\frac{\mathrm{CFU}}{\mathrm{lung}}$
*Mf* cellular interior released extracellularly to induce phagocytosis	$\upsigma$	9.2	12.7	$\frac{1}{\mathrm{d}}$
Scaling factor for *Mf*-dependent *Tf* carrying capacity	ξ	1	1	$\frac{\mathrm{lung}}{\mathrm{CFU}}$
Phagocytosis	${\mathrm{F}}_{\updelta }$	$1.7\times {10}^{-9}$	3 $\times {10}^{-9}$	$\frac{1}{\mathrm{Mac}\cdot \mathrm{d}}$
Death of *Tf* by *Mf*	${\mathrm{F}}_{\upgamma }$	1677.7	3074	$\frac{1}{\mathrm{Mac}\cdot \mathrm{d}}$
Apoptosis	${\mathrm{F}}_{{\upbeta }_{3}}$	3377	3163	1/d
Recruitment of *M*	${\mathrm{F}}_{{\mathrm{\alpha }}_{1}}$	8 $\times {10}^{6}$	6 $\times {10}^{6}$	1/d
Death of *M* by *T*	${\mathrm{F}}_{{\upbeta }_{4}}$	$6\times {10}^{-10}$	7 $\times {10}^{-11}$	$\frac{1}{\mathrm{d}\cdot \mathrm{CFU}}$
Death of *Mf* by *T*	${\mathrm{F}}_{{\upbeta }_{5}}$	0.096	0.025	$\frac{1}{\mathrm{d}\cdot \mathrm{CFU}}$
Necrosis	${\mathrm{F}}_{{\upbeta }_{6}}$	$2\times {10}^{-4}$	$1\times {10}^{-4}$	1/d
Death of *Mf*	$\widehat{{\mathrm{F}}_{{\upbeta }_{2}}}$	$168$	306	1/d

**Table 2:** Parameter adaptations (fold changes between phases).

Description	Symbol	Fold change in phase 2 respect to phase 1 value	Fold change in phase 3 respect to phase 2 value
Phagocytosis	${\mathrm{F}}_{\updelta }$	184.7	0.003
Death of *Tf* by *Mf*	${\mathrm{F}}_{\upgamma }$	313.7	0.0005
Apoptosis	${\mathrm{F}}_{{\upbeta }_{3}}$	0.4	0.005
Recruitment of *M*	${\mathrm{F}}_{{\mathrm{\alpha }}_{1}}$	6.6	0.0001
Death of *M* by *T*	${\mathrm{F}}_{{\upbeta }_{4}}$	7.2	0.147
Death of *Mf* by *T*	${\mathrm{F}}_{{\upbeta }_{5}}$	26.4	0.189
Necrosis	${\mathrm{F}}_{{\upbeta }_{6}}$	1000	102.6
Death of *Mf*	$\widehat{{\mathrm{F}}_{{\upbeta }_{2}}}$	8.7	0.3

### Five bifurcation parameters segregate clinical phenotypes into persistent infection and clearance

Previously^15^, we observed that any genotypic variant (encoded as random variations in the parameter space) resulted in one of two possible long term behaviours: bacterial clearance or invasive infection. To ask which parameters are more likely to underly this phenotypic segregation, we systematically searched for parameters that sort out the space of stable solutions into monostable and bistable (see “Methods”). The search resulted in five parameters: average $T$ phagocytized per $Mf$ ($PTf$), death of $M$ (β_1_) death of $Mf$ by $T$ (${F}_{{\upbeta }_{5}}$), recruitment of *M* (${F}_{{\mathrm{\alpha }}_{1}}$) and phagocytosis (*F*_δ_) (Fig. S1).

**Figure 2:**
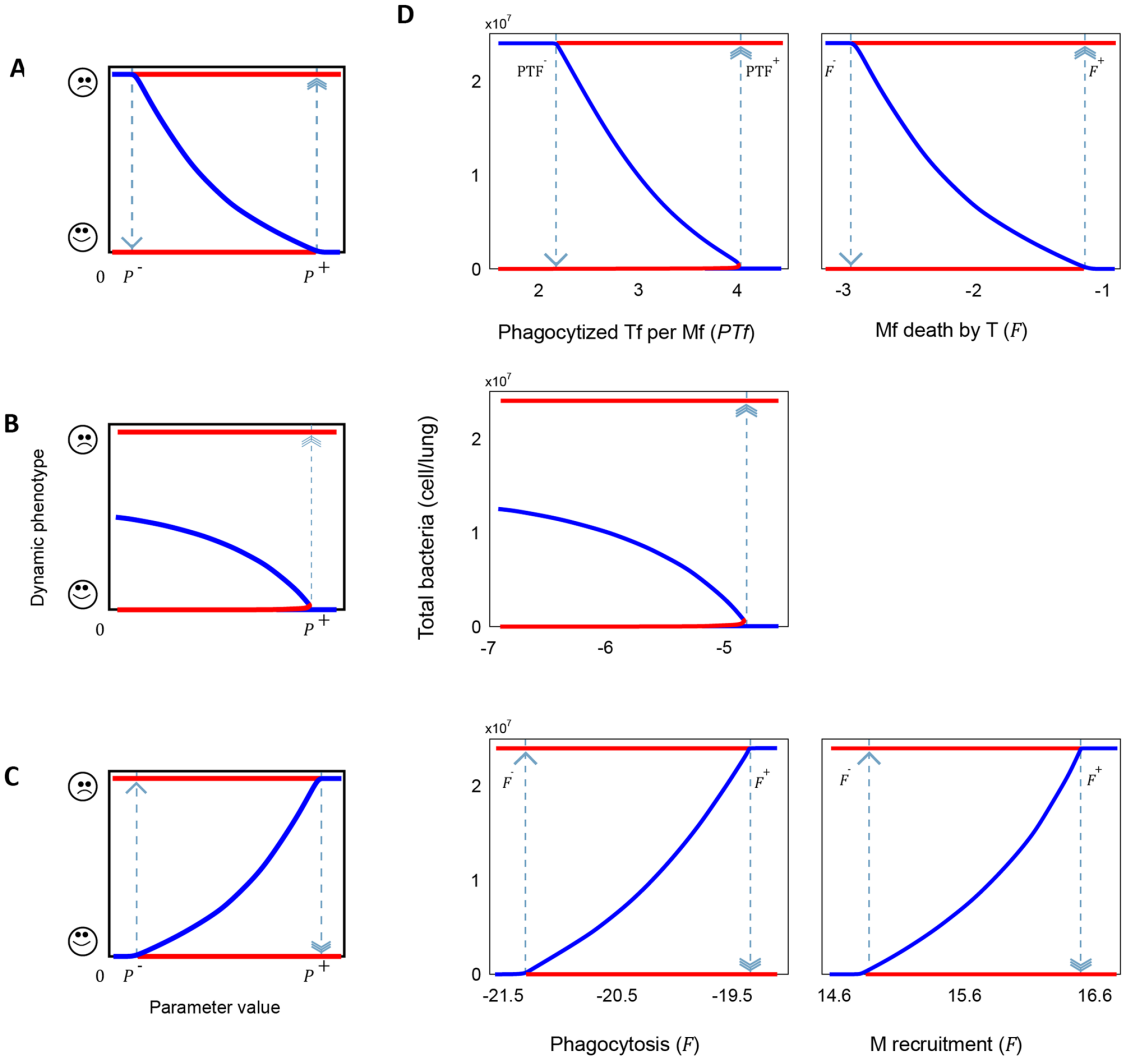
Bifurcation analysis quantitatively describes how mechanisms segregate clinical phenotypes. (**A**–**C**) Schematic representation of the three types of bifurcation diagrams found on the analysis: (**A**) Rising the bifurcation parameter to ${P}^{+}$ results in an onset of infection which prevails until the parameter is decreased to ${P}^{-}$. (**B**) Irreversible onset of infection when ${P}^{+}$ is reached. (**C**) Rising the parameter value to ${P}^{+}$ results in stable clearance of infection, which prevails unless the parameter is decreased to ${P}^{-}$. Bistability is observed in all cases between $P^{-}$ and $P^{+}$. (**D**) Bifurcation diagrams for Average $T$ phagocytized per $Mf$ (*PTf*), death of $Mf$ by *T* (${F}_{{\upbeta }_{5}}$) (*Top;* an reversible off-to-on switch as depicted in (**A**)); Death of *M* (${\upbeta }_{1}$) (*Middle;* irreversible off-to-on- switch as depicted in (**B**)), and Phagocytosis (${F}_{\updelta }$) and Recruitment of $M$ (${\mathrm{F}}_{{\mathrm{\alpha }}_{1}}$) (*Bottom;* irreversible on-to-off switch as depicted in (**C**)). Red lines represent asymptotically stable solutions and blue lines are unstable solutions. Dotted vertical arrows are the threshold parameter values when an abrupt change in stability occurs (i.e., a bifurcation). Parameter values in the x-axis are shown in log10 scale.

Next, we performed bifurcation analyses on these five parameters, by varying the values around their Ph1-specific nominal value (Table 1) on a one-by-one basis. For all parameters, the resulting bifurcation diagrams show a switch-like dose response curve, with two possible stable equilibrium points corresponding to uncontrolled infection and bacterial clearance (red lines on Fig. 2). All five bifurcation diagrams corresponding to the *i* = [1,…,5] bifurcation parameters ${P}_{i}$ show a bistable region $[{{P}_{i}}^{-}{, {P}_{i}}^{+}]$ in which these two scenarios stably coexist.

The five bifurcation diagrams for Ph1 show three distinct dynamical behaviors (Fig. 2A–C).

The first class of bistable bifurcation diagram shows an abrupt transition from bacterial clearance to persistent infection when the bifurcation parameter is increased to ${P}_{i}^{+}$. Once surpassed this threshold, long term recovery can only be achieved if ${P}_{i}$ is decreased below ${P}_{i}^{-}$ (Fig. 2A). This behavior is shown by the bifurcation parameters: Average $T$ phagocytized per $Mf$ ($PTf$) and death of $Mf$ by $T$ (${F}_{{\upbeta }_{5}}$). The bifurcation diagram for $PTf$ shows bacterial clearance for values below $PT{f}^{-}\sim$ 9 CFU/Mac and persistent infection when above $PT{f}^{+}$~61 CFU/Mac (Fig. 2D, top), suggesting that bacteria benefit from being phagocytized in large numbers. Consistent with our predictions, live cell imaging has demonstrated that the number of *Mtb* internalized by individual cells determines macrophage fate; phagocytosis of large aggregates is more cytotoxic than multiple small aggregates containing similar numbers of bacilli^30^. The second bifurcation parameter leading to such abrupt recovery-to-persistence behavior is ${F}_{{\beta }_{5}}$. A higher rate of killing of phagocytic macrophages by free bacteria causes the bacterial population to thrive (Fig. 2D, top right). This result is consistent with recent observations suggesting that extracellular *Mtb* aggregates can evade phagocytosis by killing macrophages in a contact-dependent but uptake-independent manner^31^. Also, *Mtb* can secrete bacterial membrane vesicles containing toxins, virulence factors, nucleic acids, and other molecules that affect the phagocytic capacity of host cells^32^.

In the second type of behavior, increases in the bifurcation parameter beyond ${P}_{i}^{+}$ result in an abrupt and irreversible transition from bacterial clearance to persistent infection, as the value for recovery ${P}_{i}^{-}<0$ and hence biologically unattainable (Fig. 2B). This behavior emerges from varying the death rate of $M$ (β_1_), while its value increases, chances of bacteria reaching its carrying capacity also do so (Fig. 2D, middle). A macrophage population with a shorter lifespan means a slower accumulation of macrophages at the site of infection, resulting in a reduced antimicrobial force. As ${\beta }_{1}$ encapsulates multiple macrophage death mechanisms, including efferocytosis, autophagia, necroptosis and pyroptosis -which have been shown to vary across resident alveolar macrophages and inflammatory macrophages and the microenvironment conditions they’re subject^33–37^, it is plausible to think that this parameter can vary even orders of magnitude around the nominal value. Indeed, it has been experimentally shown that, in the context of a mouse model of pulmonary TB, macrophage death can increase up to two orders of magnitude when the highly virulent Beijing strain 9,501,000 is used^38, 39^. Further experiments are required to determine whether differences in host condition can lead to even larger variation in macrophage death rates.

Finally, in the third type of bifurcation diagram, an inverse relation between parameter value and disease severity is observed. Here, increasing the parameter value up to ${P}_{i}^{+}$ results in a transition from persistent infection to remission. Once in remission, decreasing the parameter value below ${P}_{i}^{-}$ results again in a persistent infection (Fig. 2C). This behavior is displayed by the rates of phagocytosis (*F*_δ_) and of recruitment of *M* (${F}_{{\mathrm{\alpha }}_{1}}$) (Fig. 2D, bottom). Our results show that a higher phagocytosis rate leads to bacterial clearance. As the only mechanism of bacteria eradication in our model is intracellular, ${F}_{\updelta }$ is strictly necessary for infection control. There is a wide range of phagocytic receptors that interact with a variety of phagocytosis signaling cascades, and receptors have various degrees of ligand specificity, making ${F}_{\updelta }$ a modulable mechanism^40^. Mycobacteria display numerous and diverse ligands on their surface which can engage with multiple macrophage receptors of multiple types simultaneously^41^. Augmenting ${F}_{{\mathrm{\alpha }}_{1}}$ also leads to bacterial clearance. As with ${\beta }_{1}$, this mechanism is arguably vital for maintenance of macrophage population at the site of the infection and subsequently crucial for controlling bacterial growth. Nevertheless, a high and persistent macrophage recruitment could result in excessive inflammation, which has been shown to have counter effects for the host^42, 43^. A combination of increased macrophage efficiency and controlled inflammation could result in an efficient and non-toxic treatment.

### Disease phase affects the sensitivity of the clinical phenotype to changes in the bifurcation parameters

**Figure 3:**
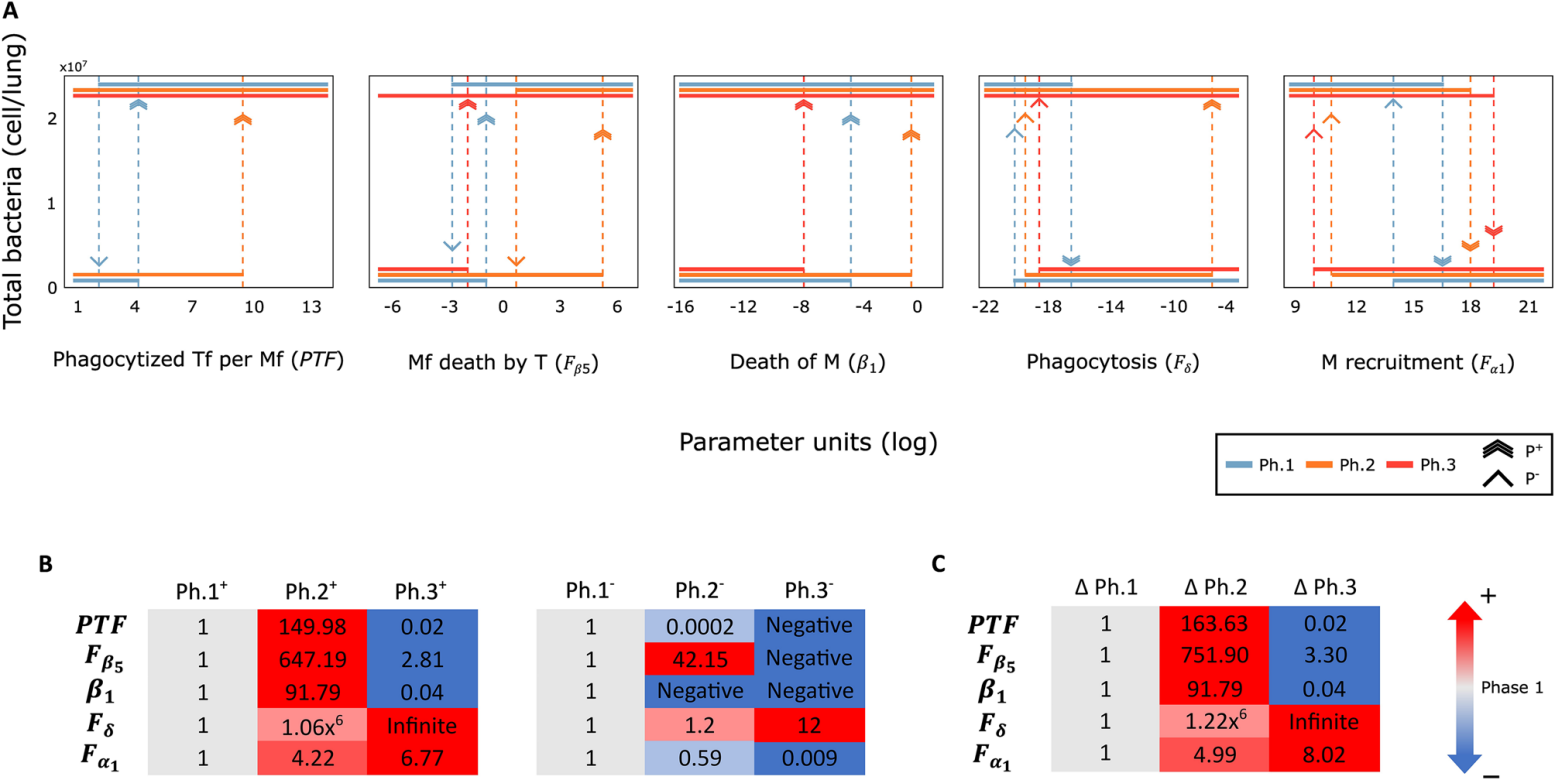
Disease progression affects the sensitivity of the clinical phenotype to changes in the bifurcation parameters. (**A**) Bifurcation diagrams for the five bifurcation parameters across the 3 phases. Unstable solutions are not shown for clarity. Dotted vertical arrows denote threshold parameter values when an abrupt change in stability happens, from bacterial clearance to invasive infection or vice versa. (**B**) Right (*P*^−^) and left (*P*^+^) threshold parameter values enclosing the bistable region change as the disease progresses. Each row corresponds to one of the 5 bifurcation parameters, and the columns are the *P*^−^ (left panel) *P*^+^ (right panel) threshold parameter values for each phase, normalized to its phase 1—specific threshold value. (**C**) Size of the bistable region. For negative *P*^−^, Δ*P* corresponds to *P*^+^.

Following the Ph1 analysis, we studied how disease progression affects the bifurcations. For this, we conducted a similar bifurcation analysis as in the previous section but this time varying the parameters around their specific Ph2 and Ph3 nominal values (Table 2). While a switch-like bistable bifurcation diagram is observed across all phases and for all bifurcation diagrams of the model, progression from Ph1 to Ph2 and to Ph3 dramatically shifts the threshold values ${P}^{-}$ and ${P}^{+}$ (Fig. 3).

For $PTf$, ${F}_{{\upbeta }_{5}}$ and ${\beta }_{1}$, ${P}^{+}$ increases from Ph1 to Ph2 and decreases to its lowest value in Ph3, while for ${F}_{\updelta }$ and ${F}_{{\mathrm{\alpha }}_{1}}$ this threshold has a staggered growth through the three phases. For ${F}_{\updelta }$, ${P}^{-}$ is also constantly increasing indicating that the bistable window gets shifted to the right. In contrast, ${P}^{-}$ in ${F}_{{\mathrm{\alpha }}_{1}}$ decreases across the phases, which means that the bistable region keeps growing steadily through the phases (Fig. 3C). For ${F}_{{\upbeta }_{5}}$ and $\mathrm{PT}f$, ${P}^{-}$ < 0 in Ph3, causing a shift from a reversible to an irreversible switch.

For $PTf$, the switch becomes irreversible from Ph2, meaning that a therapeutic intervention through decreasing this mechanism is exclusive to Ph1. It is worth pointing out that $PT{{f}^{+}}_{Ph.2}\gg PT{{f}^{+}}_{Ph.1}\gg PT{{f}^{+}}_{Ph.3}$, suggesting that M1 macrophages can internalize many bacteria without necessarily leading to persistent infection. Clinical evidence shows that thanks to the adaptive immune response most infected individuals contain the bacilli and never develop active disease^44^. The bistable region for ${F}_{{\upbeta }_{5}}$ gets pushed to the right several orders of magnitude in Ph2, resulting in an ideal timing for therapeutic interventions over Ph1. In Ph3 the switch becomes irreversible, hence bacterial clearance can no longer be achieved by tuning *Mf* death by *T,* and therefore a treatment focused on this mechanism would be exclusive to patients on the first two phases of disease progression.

For ${\beta }_{1}$ the switch remained irreversible across the three phases, with the position of its right (and only) threshold dramatically increasing by three orders of magnitude between Ph1 (${{{\beta }_{1}}^{+}}_{Ph1}=0.00719$) and Ph2 (${{{\beta }_{1}}^{+}}_{Ph2}=0.66$). This threshold displacement indicates that Ph2 is less vulnerable to increased macrophage mortality, suggesting that activated macrophages are better at controlling the infection even when their lifespan is shortened. This robustness is lost in Ph3 (${{{\beta }_{1}}^{+}}_{Ph3}=0.0003$), showing again the increased efficacy of M1 macrophages over M2 and non-activated ones.

For ${F}_{\updelta }$, the switch remains bistable with a larger hysteresis region during Ph2 (${{\Delta F}_{\updelta }}^{Ph1}={3.45}^{-9}$, ${{\Delta F}_{\updelta }}^{Ph2}={4.2}^{-3}$) but becomes irreversible for Ph3. Further, during Ph3, ${{F}_{\updelta }}^{+}$ increases far beyond an attainable value (above 31 orders of magnitude of the nominal value), suggesting that targeting the strength of phagocytosis to achieve bacterial clearance is only possible for the first two phases. This large phase-dependent variation in the sensitivity of the infection outcome to changes in the phagocytosis rate could be explained by changes in the metabolic activity of M1 and M2 macrophages. Experimental manipulation of the hypoxia-inducible factor-1 alpha (HIF-1a) can dramatically affect the infection outcome, by interfering with the metabolic reprogramming that characterizes M1 to M2 transitions^45^, while simultaneously affecting the phagocytic capacity of macrophages^46^. It remains to be elucidated whether other treatments such as mycobacterial proteins P27 and PE-PGRS33, which affect intercellular bacillary load in alveolar macrophages^47^, are more effective during the early phases of infection.

Finally, ${F}_{{\mathrm{\alpha }}_{1}}$ showed a reversible switch for the three phases, with ${F}_{{\mathrm{\alpha }}_{1}}>{{F}_{{\mathrm{\alpha }}_{1}}}^{+}$ leading to bacteria eradication. As the disease progresses ${{F}_{{\mathrm{\alpha }}_{1}}}^{+}$ increases, requiring a more aggressive therapy for clearing the infection. Increase in ${{F}_{{\mathrm{\alpha }}_{1}}}^{+}$ is mirrored by an increase in the length of the bistability region ($\Delta {F}_{{\mathrm{\alpha }}_{1}}$), suggesting that once bacterial clearance has been achieved, this phenotype is more robust to decreases in macrophage recruitment. Nevertheless, a therapeutic intervention of a Ph3 patient would be the most difficult due to the risk of excessive inflammation.

Overall, the results above show the importance of considering the disease phase when designing a therapeutic intervention in progressive diseases.

### Two-dimensional bifurcation analysis reveals crosstalk among mechanisms.

**Figure 4:**
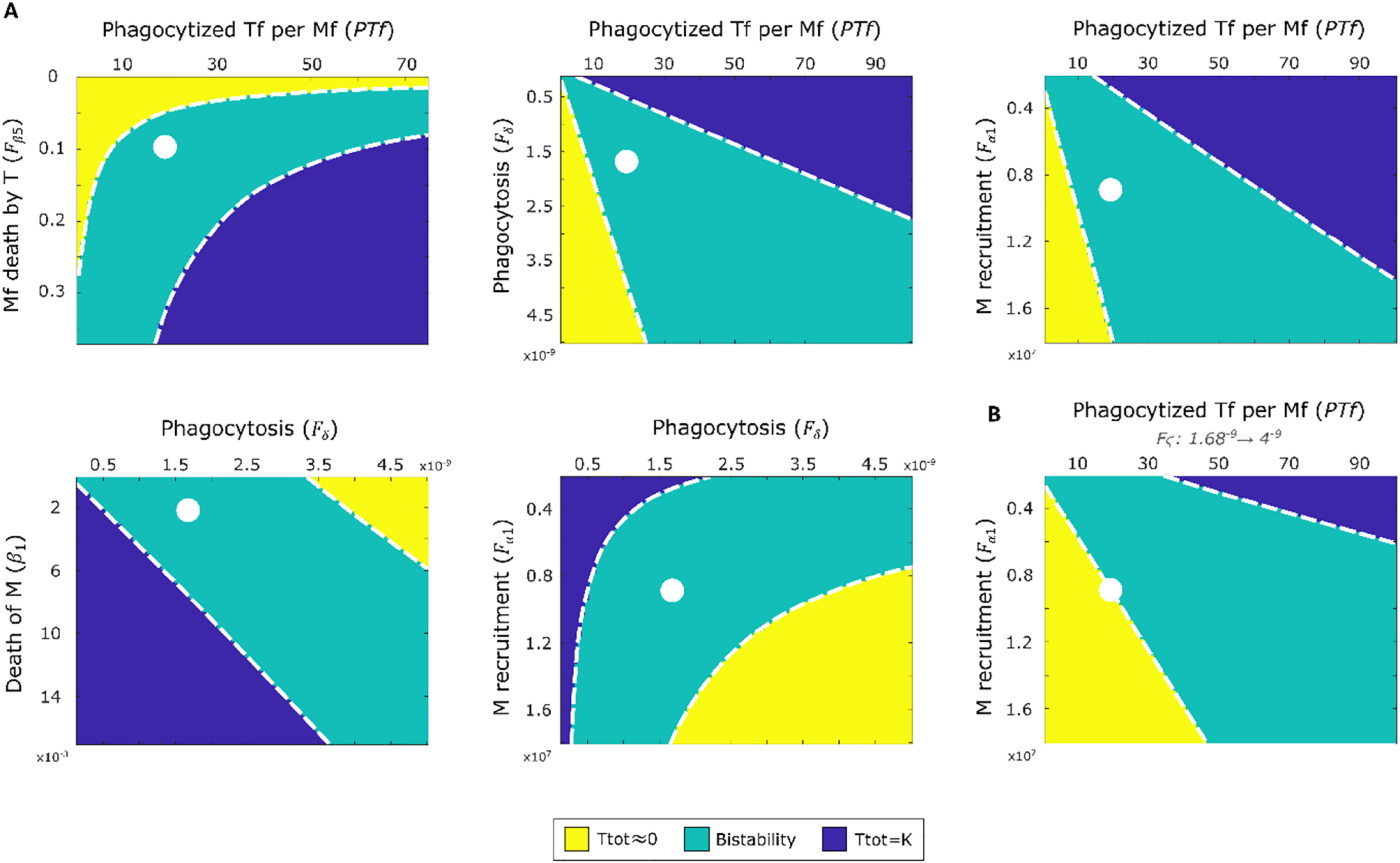
Two-dimensional bifurcation analysis exposes synergistic and antagonistic effects of compound regulation for two (**A**) and three (**B**) parameters simultaneously. Dotted white lines are $({P}_{\mathrm{i}}^{-}, {P}_{\mathrm{j}}^{-})$  and $(P_{\mathrm{i}}^{+}, P_{\mathrm{j}}^{+})$ bifurcation curves that delimitate the bistable space. Nominal Ph1 parameter values (Table 1) are shown as white dots.

To analyze the dynamic interplay between pathophysiological mechanisms, we performed bifurcation analysis varying two parameters simultaneously around their Ph1 specific nominal values (Table 1). Each panel on Fig. 4 shows a 2-dimensional bifurcation diagram with the bistable region (cyan) limited by the bifurcation curves ($${P}_{i}^{-}, {P}_{j}^{-})$$ and ($${P}_{i}^{+}, {P}_{j}^{+})$$, as well as the mono-stable regions for bacterial clearance (yellow) and for persistent infection (blue). While all possible combinations of the five bifurcation parameters were performed (Supplementary Fig. 4), here we show only the ones with the biggest potential for treatment design.

The pair *P*_*i*_*: # T* phagocytized by *M* ($$PTf$$) $$\times$$ *P*_*j*_: Death of *Mf* by *T* ($${F}_{{\upbeta }_{5}}$$) shows synergistic effects, since the corresponding bifurcation curves ($${P}_{i}^{-}, {P}_{j}^{-})$$ and ($${P}_{i}^{+}, {P}_{j}^{+})$$ show that there is a non-additive relation between these two parameters. We see bacterial clearance at all values of $$PTf$$ when $${F}_{{\upbeta }_{5}}$$ is below 0.0158. This contrasts with the 1-dimensional analysis (Fig. 2A) showing persistent infection when $$PTf>61\mathrm{ CFU}/\mathrm{Mac}$$. Similarly, $${F}_{{\upbeta }_{5}}$$ alone leads to persistent infection when above 0.32 $$\frac{1}{CFU\cdot d}$$ but remains bistable if $$PTf$$ is small enough. These results suggest that macrophages can deal with greater amounts of internalized bacteria if the extracellular activity of the pathogen is reduced.

The pair *P*_*i*_*:* # *T* phagocytized by *M* ($PTf$) $\times$ *P*_*j*_: Phagocytosis ${(F}_{\delta }$) shows a linear but opposite relation. While large values of $PTf$ at low ${F}_{\delta }$ results in unresolved infection, increasing ${F}_{\delta }$ can result in bacterial clearance even for high $PTf$. This suggests that a higher phagocytosis rate could compensate for a possible rise in $PTf$ due to bacteria-induced alterations of the macrophage. Augmenting ${F}_{\delta }$ could therefore be an effective nontoxic therapeutic approach; indeed, phagocytosis has been shown to have a central role in the resolution of inflammation^36^.

Similarly, the pair *P*_*i*_*:* # *T* phagocytized by *M* ($PTf$) $\times$ *P*_*j*_: recruitment of *M* (${F}_{{\mathrm{\alpha }}_{1}}$) shows a linear but opposite relation. A transition from persistent infection to clearance can be achieved if $PTf$ is reduced or if ${F}_{{\mathrm{\alpha }}_{1}}$ is increased; and if $PTf$ is sufficiently small, then smaller increases in ${F}_{{\mathrm{\alpha }}_{1}}$ are sufficient for a transition to a healthy phenotype. In vitro experiments show that macrophages internalize 7 mycobacterium per cell in average^27^, making a $PTf$ reduction strategy plausible and more convenient than rising ${F}_{{\mathrm{\alpha }}_{1}}$ which can cause inflammatory damage.

Along the same line, the *P*_*i*_*:* Phagocytosis ${(F}_{\delta }$) $\times$ *P*_*j*_: Death of *M* (${\upbeta }_{1}$) bifurcation diagram shows a linear but opposite relation between these two mechanisms. While increasing the phagocytosis rate can lead to effective bacterial clearance, macrophage death rate must be small enough, otherwise even high values of ${F}_{\delta }$ will result in persistent infection.

The *P*_*i*_*:* Phagocytosis ${(F}_{\delta }$) $\times$ *P*_*j*_: Recruitment of *M* (${F}_{{\mathrm{\alpha }}_{1}}$) bifurcation diagram shows a synergistic relation between these two mechanisms. Our analysis suggests that, while increasing either of these rates alone could result in bacterial clearance, increasing both simultaneously would dramatically reduce the intervention effort; smaller increases would be sufficient to achieve remission.

Figure 4B shows the *P*_*i*_*:* # *T* phagocytized by *M* ($PTf$) $\times$ *P*_*j*_: recruitment of *M* (${F}_{{\mathrm{\alpha }}_{1}}$) diagram when the value of ${F}_{\delta }$ is raised. The bistable area increases, resulting in a smaller ${F}_{{\mathrm{\alpha }}_{1}}$ value (up to one order of magnitude) needed to converge to the healthy phenotype. This suggests that phagocytosis can drive bacterial clearance without higher levels of inflammation.

In summary, our analysis predicts different types of combinatorial effects between pathogenic mechanisms. Of particular relevance are the synergistic parameter pairs ($PTf\times {F}_{{\upbeta }_{5}}$ and ${F}_{\updelta }\times {F}_{{\mathrm{\alpha }}_{1}}$), where a desired clinical phenotype can be achieved with less treatment effort when both mechanisms are modulated simultaneously than when they are varied one at the time. Thus, these pairs could constitute particularly promising unconventional therapeutic targets.

### Modulation of speed of transition across disease phases

**Figure 5:**
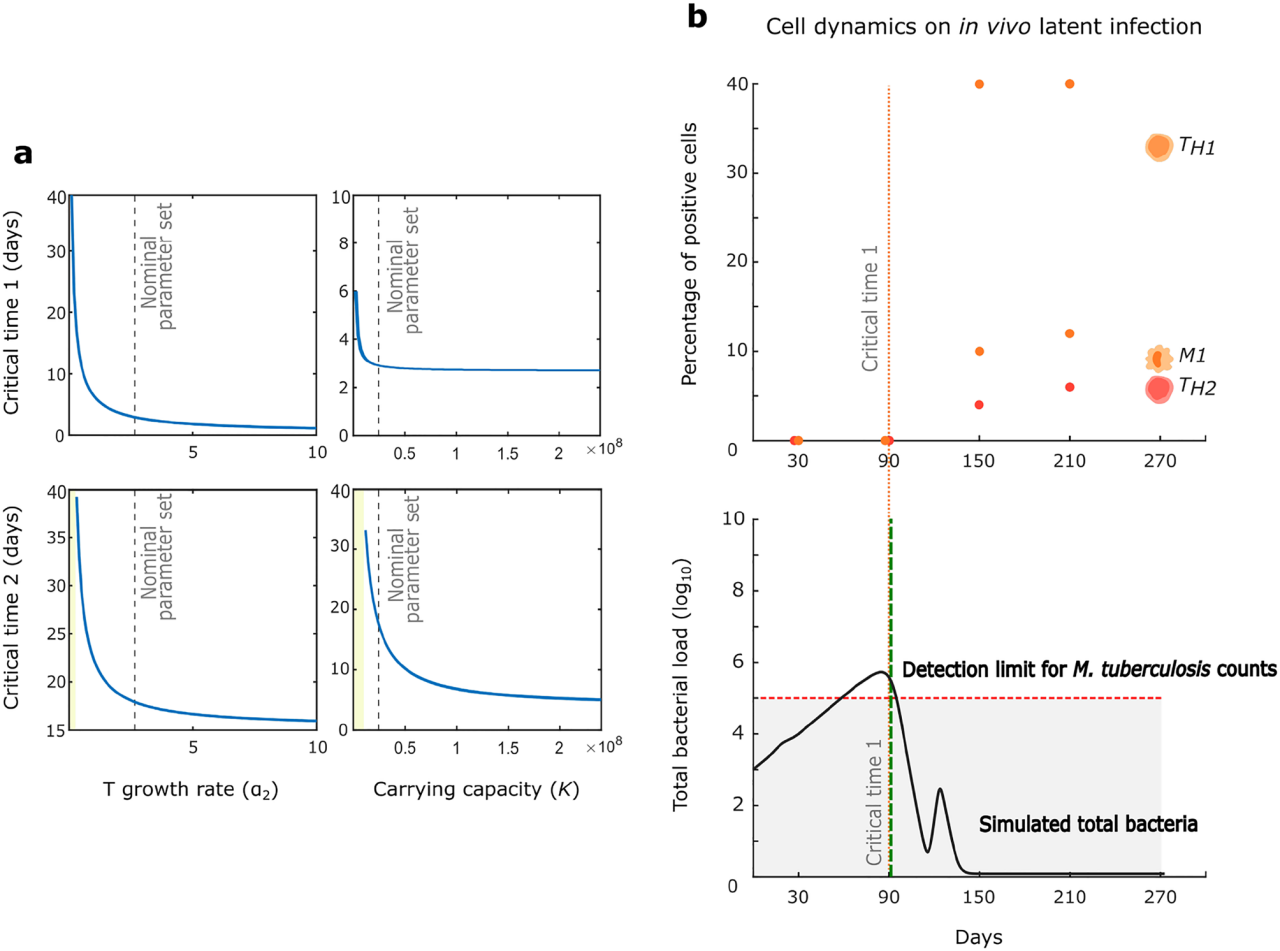
Parameter variation affects the transitory state of the model. (**a**) Variations in *T* growth rate (α_2_) and in bacterial carrying capacity (*K*) affect the speed of progression across phases. (**b**) Model captures latent tuberculosis infection (LTBI) cellular dynamics. *Top*: Phase 2-specific cellular markers Th1, Th2 and M1 macrophages rise sharply at the critical time 1 = 90 days post infection in a B6D2F1 mouse model of LTBI (data from^53^). *Bottom:* Mathematical model reproduces the mouse model of latent tuberculosis infection described in (Arriaga et al.^53^); the simulated total bacterial loads remain below the limits of detection for almost all the course of the simulation, and the simulated critical time 1 for onset of adaptive immune responses (dotted vertical green line) coincides with the corresponding experimental value (dotted vertical orange line spanning from top to bottom panel) from^53^.

A key aspect of our model is that the disease is not stagnant in a specific phase, but rather transitions between them dynamically. Hence, we explored how parameter variations modulate the speed of transitions across phases. This is important to better design the timing of treatments, e.g., if a treatment works best in Ph2, we want to know how long it will take a given patient to arrive at that phase, and how long it will last. To study this, we quantified the impact of variation of all model parameters on the critical times at which the disease progresses from Ph1 to Ph2 (critical time 1) and from Ph2 to Ph3 (critical time 2). Among all parameters, only the growth rate of free bacteria (${\alpha }_{2}$) and the carrying capacity ($K$) show a significant effect on these critical times (Fig. 5); interestingly, none of the bifurcation parameters had a major effect on these transition times (Supplementary Fig. 3). This is consistent with experimental results, showing that the single most important factor affecting the phase transition speed is the bacterial growth kinetics, which in turn depends on the specific bacterial strain^48^ and on endocrine factors of the host^49^.

Parameter ${\alpha }_{2}$ showed a strong influence on critical time 1 (Fig. 5a), peaking at 39 days for ${\alpha }_{2}=0.1$ and quickly decreasing up to 9 days for ${\alpha }_{2}=0.6$. A similar trend is observed for critical time 2, with a rapid decrease from 39 days at ${\alpha }_{2}=0.3$ to a minimal value of 16 days at ${\alpha }_{2}=5.7$. For values equal and less than 0.2, no transition to Ph3 is observed. As shown in the previous section, therapeutic interventions have a strong dependence on time. This means that patients infected with highly virulent strains of *M. tuberculosis*, which have higher ${\alpha }_{2}$ rate^6, 48^ could have a shorter window for successful treatment intervention. Parameter $K$ has a minor effect on critical time 1 but strongly affects critical time 2 (Fig. 5a). None of the two parameters showed bifurcations (Supplementary Fig. 3).

Manipulation of parameter values that affect transition times allowed us to reproduce the disease dynamics observed in latent TB infections (LTBI). A latent TB infection is defined as a state of persistent immune response against *M. tuberculosis* antigens without evidence of clinical manifestation of active TB disease^50^. Due to the low number of bacteria in the tissue in latent infections, the clinical diagnosis of these does not focus on the identification of the bacteria or their components but on their indirect detection through evidence of cellular immune response to bacterial antigens^51^. In vivo models of chronic infection similar to latent infection in mice^56^ show the appearance of Th1 lymphocytes and the subsequent activation of M1 macrophages 90 days after infection^53^ (Fig. 5b), that is, more than 80 days later than in a progressive infection^54, 55^. This proinflammatory adaptive immune response is maintained throughout the LTBI assay in^53^ without transitioning into a Th2 recruited anti-inflammatory response. From the above, we can conclude that a latent infection has a Ph1 to Ph2 transition but not to Ph3. Hence, we can interpret latent infection as Ph2 that converges ultra-slowly towards bacterial clearance or persistent infection (i.e., the critical time 2 $\to \infty$). Although detection of bacteria by culture should be impossible in latent infections (detection limit shown as a dotted line in Fig. 5b), the in vivo model of^53^ conforms to the operational definition of an experimental latent infection consisting of a stable population of bacteria in the tissue without the presence of symptomatic disease in a long period of time and without spontaneous reactivation of the infection^52^.

Our model was able to mimic bacterial population dynamics in the first 90 days and replicates the recruitment time of the adaptive immune response (critical time 1) and the non-recruitment of the anti-inflammatory response. This was achieved by decreasing the initial numbers of free bacteria in the system, which is consistent with the lower bacterial challenge used in LTBI assays vs. progressive infection ones. Additionally, all the Ph1 parameters of the system were varied slightly (Table 1), to mimic the difference in the genetic background used for the LTBI (B6D2F1 mouse strain) with respect to the progressive infection model (BALB/c strain) used for the original calibration of the data. Consistent with our theoretical results (Fig. 5a), parameter ${\alpha }_{2}$ had to vary by one order of magnitude from its nominal value to reproduce the higher critical time 1 observed in the LTBI.

Subsequent in silico experiments (Supplementary Fig. 2) show that this convergence is even slower than the maximum lifespan of a mouse under laboratory conditions^56^, suggesting that a latent infection will not reactivate unless there is an external forcing (sudden immunocompromise, old age, etc.).

## Discussion

Here we presented a quantitative characterization of the influence of specific disease mechanisms on the clinical outcome of *Mtb* infection, which we achieved through bifurcation analysis of our minimal mathematical model of tuberculosis progression. Our results pinpoint five specific mechanisms and report how changes in their magnitude secern genotypes (parameter sets) into clinically relevant phenotypes (stable solutions). Additionally, we characterized the influence of disease progression on the sensitivity to variations in these key mechanisms, exposing the importance of the disease stage in the mechanisms’ ability to determine disease outcomes. We also revealed synergic effects between disease mechanisms through 2-dimensional bifurcation analyses. Finally, we identified the model parameters that control the pace of phase transitions, and with this, could reproduce latent tuberculosis infection.

Our bifurcation analysis suggests that the number of bacteria a macrophage accumulates is crucial to its survival, a prediction that is supported by experimental data^30^. There are two ways for *Mtb* to accumulate inside macrophages: By trapping macrophages into phagocytizing multiple bacteria simultaneously or through converting macrophages into permissive niches. According to^30^, *Mtb* aggregate naturally unless the medium is transformed, for example by using a non-ionic detergent such as Tween 80. A non-toxic way to transform the host’s extracellular environment could be explored as a therapeutic approach. On the other hand, *Mtb’s* ability to block phagosome maturation, evade autophagy and manipulate the inflammasome turns macrophages into bacteria reservoirs^44^ which will continue to phagocytose without eliminating already internalized bacteria until reaching a potentially lethally high $PTf$. Besides known antibiotics like Rifampicin and Ethambutol than can eliminate bacteria inside or outside macrophages^57^, novel immunotherapies based on antimicrobial peptides that do not depend on bacterial replication (like conventional antibiotics do) have been proved to reduce pulmonary bacillary loads in TB infection^58^. Experimental evidence^31^ shows yet another risk caused by bacteria aggregation, namely, the capacity of *Mtb* aggregates to kill macrophages extracellularly. Our predictions on the dangerous consequences of rising *Mf* death by *T* reaffirm the necessity to stop bacteria from grouping in big numbers on the extracellular environment. Our results also suggest that increased macrophage death can result in an uncontrolled infection, the greatest susceptibility to these fluctuations being in phase 3. Chemotherapeutic or other pharmacological regimes^59^ as well as aging^60^ can alter the lifespan of alveolar macrophages, making those patients vulnerable to developing an aggressive symptomatic disease if infected with *Mtb*. Our results suggest that increasing phagocytosis and macrophage recruitment can lead to a favorable outcome, and that the manipulation effort to achieve this goal increases as disease progresses. Due to the multiple evidence pointing that an excessive inflammation is highly detrimental to the organism^42, 61^, a therapy focused on promoting phagocytosis could be safer than rising macrophage recruitment. Recent studies have demonstrated that silencing the transcriptional factor Klf10 in macrophages promotes bacterial clarification by augmenting IFN-$\upgamma$ levels which boosts phagocytosis and bacilli destruction^62^.

Tuberculosis is a progressive disease that displays multiple facets of the immune system. Here, we studied the influence of disease progression on the susceptibility of the host to modulations in the parameter strength driving the clinical outcome of the infection. For ${\beta }_{1}$, ${F}_{{\mathrm{\alpha }}_{1}}$ and ${F}_{{\upbeta }_{5}}$, it is during Phase 2 that less effort is required to achieve bacterial clearance; further, the host is most robust to persistent infection. Immunotherapies that regulate the host response rising macrophage activation, recruitment, phagocytic and antimicrobial capability using adenovirus based-vectors to produce antimicrobial peptides like catheldicins and defensins and pro-Th1 cytokines like IFN-$\upgamma$ and IL-12 have been proposed^63, 64^. Driving the organism to a Phase 2-like phenotype through these immunotherapies will make ${\beta }_{1}$, ${F}_{{\mathrm{\alpha }}_{1}}$ and ${F}_{{\upbeta }_{5}}$ manipulation more efficient. It is important to keep in mind that certain comorbidities like AIDS and diabetes present a dramatic decrease in CD4^+^ T-cells^65^, making it almost impossible for an adaptive immune response to be recruited. Parameters ${F}_{\delta }$ and $PTf$ showed that a therapeutic intervention through these mechanisms is only possible in Phase 1, highlighting the importance of early detection and treatment of TB. Only ${\beta }_{1}$ and ${F}_{{\mathrm{\alpha }}_{1}}$ were able to lead the system to bacteria clearance on Phase 3, this phase being the one with the highest parameter thresholds for remission (i.e., more resource consuming). Experimental evidence shows that blocking cytokines like IL-4 and IL-10 that play an anti-inflammatory role in late active TB may constitute an efficient therapeutic strategy^66^.

Two-dimensional bifurcation analysis revealed synergic effects between mechanisms, showing how compound manipulation could lead to a more efficient therapeutic approach. In the ${F}_{\updelta }\times {F}_{{\mathrm{\alpha }}_{1}}$ diagram, a synergistic effect was found when both parameters increased simultaneously, requiring almost half the recruitment needed to drive the system to bacterial clearance when ${F}_{\delta }$ is raised by two units, reducing the risk of excessive inflammation. An immunotherapy that increases IFN-$\upgamma$^62^ and blocks IL-4 and IL-10 cytokines^66^ will increase ${F}_{\delta }$ and prevent ${F}_{{\mathrm{\alpha }}_{1}}$ from decreasing, resulting in an efficient nontoxic therapy. Phagocytosis and recruitment synergy also showed to work even in scenarios where $PTf$ rises. The other synergy observed was between $PTf\times {F}_{{\upbeta }_{5}}$, demonstrating that healthy macrophages can bear large intracellular bacilli loads. To achieve the goal of less toxic and less resource consuming therapies, crosstalk among mechanisms must be considered.

In addition to the model mechanism’s ability to determine dynamic phenotypes, they also influence its transient state. While it is essential to obtain the equilibrium behavior of the system, the path followed to get there is equally important, especially in terms of resource efficiency. While none of the 5 bifurcation parameters alter the transition times between phases, ${\alpha }_{2}$ and $K$ are capable of dramatically accelerate disease progression as their values increase. Variation in bacteria proliferation levels has been documented through different strains of *M. tuberculosis*, Strain 09005186 produces twice the CFU at 21 days post-infection in BALB/c mice than our nominal H37Rv model strain^48^. Strain 09005186’s higher ${\alpha }_{2}$ would result in quicker phase transitions, narrowing the time windows for efficient treatment, hence knowing *Mtb* strain is fundamental for effective therapy design. On the other hand, ${\alpha }_{2}$ values closer to zero would lead to ultra-slow disease progression, which is a hallmark of latent tuberculosis infection. LTBI is the most persistent infection state worldwide^51^, which we could mathematically replicate as an ultra-slow Ph2 that will take longer to converge than the lifespan of organisms.

Our model is deterministic, and hence, its main limitation is that it reproduces only the mean field behaviors of the macrophage and bacteria populations. A stochastic version of our model that includes intrinsic noise in the individual reactions as well as extrinsic noise in rate constants would allow us to simulate a distribution of heterogeneous phenotypes, and to explore how the shape of these distributions could be modulated by therapeutic interventions. Additionally, while in our model we make the simplifying assumption that bacterial killing by macrophages is only intracellular, it is plausible that extracellular mechanisms also shape the disease outcome. Finally, phagocytosis was modeled with a Gause-type approach, but alternatively, a Leslie-Gower formulation might be explored^67^.

Future work involves the recalibration of our model with clinical longitudinal data to attain translational medicine^68^ and to replicate the synergy between antibiotics and immunotherapy that has been observed experimentally^64, 69^, as previously done for *S. pneunomiae* infection^7^.

The five bifurcation parameters found correspond to mechanisms that are predicted by our model to determine the outcome of TB infection. Therapeutic interventions targeting these single mechanisms must contemplate disease progression stage as well as the state of the other mechanisms (genetic and microenvironmental backgrounds). Through bifurcation analysis, our model proves to be a powerful tool to quantitatively assess the influence of its mechanisms on the determination of the dynamic phenotype, opening the door to further in silico experimentation that will lead to the design of experiments and new efficient treatments.

## Methods

### Model equations

The model of TB progression (Fig. 1A) represents the dynamical coupling between free macrophages ($M$*),* macrophages containing phagocyted *Mycobacterium tuberculosis* ($Mf$), free *Mycobacterium tuberculosis* ($T$) and phagocyted *Mycobacterium tuberculosis* ($Tf$) as:

1 $$\dot{M} = \overbrace {{Mf \cdot F_{\alpha 1} }}^{{{\text{Recruitment}}}} - \overbrace {{M \cdot \frac{T}{PTf} \cdot F_{\delta } \cdot \left( {1 + Mf \cdot F_{\beta 6} \cdot \sigma } \right)}}^{{{\text{Phagocytosis}}}} - \overbrace {{M \cdot \beta_{1} }}^{{{\text{Death}}}} - \overbrace {{M \cdot T \cdot F_{\beta 4} }}^{{{\text{Death by }}T}},$$

2 $$\dot{M}f = \overbrace {{M \cdot \frac{T}{PTf} \cdot F_{\delta } \cdot \left( {1 + Mf \cdot F_{\beta 6} \cdot \sigma } \right)}}^{{{\text{Phagocytosis}}}} - \overbrace {{Mf \cdot T \cdot F_{\beta 5} }}^{{\text{Death by T}}} - Mf \cdot \left( {\overbrace {{\widehat{{F_{\beta 2} }}}}^{{{\text{Death}}}} + \overbrace {{F_{\beta 3} }}^{{{\text{Apoptosis}}}} + \overbrace {{F_{\beta 6} }}^{{{\text{Necrosis}}}}} \right),$$

3 $$\dot{T} = \overbrace {{\alpha_{2} \cdot T \cdot \left( {1 - \frac{T}{K}} \right)}}^{{{\text{Proliferation}}}} + \overbrace {{Mf \cdot F_{\beta 6} \cdot PTf}}^{{{\text{Necrosis}}}} - \overbrace {{M \cdot \frac{T}{PTf} \cdot F_{\delta } \cdot \left( {1 + Mf \cdot F_{\beta 6} \cdot \sigma } \right)}}^{{{\text{Phagocytosis}}}},$$

4 $$\dot{T}f = \overbrace {{\alpha_{3} \cdot Tf \cdot \left( {1 - \frac{Tf}{{\left( {1 + \tilde{K} \cdot Mf\cdot \xi } \right)}}} \right)}}^{{{\text{Proliferation}}}} + \overbrace {{M \cdot \frac{T}{PTf}F_{\delta } \cdot \left( {1 + Mf \cdot F_{\beta 6} \cdot \sigma } \right)}}^{{{\text{Phagocytosis}}}} - \overbrace {{Mf \cdot Tf \cdot F_{\gamma } }}^{{{\text{Death by}} Mf}}.$$

This is a system of four ordinary differential equations (ODEs) where the ‘dot’ in $\dot{M}=\frac{dM}{dt}$ represents the rate of change of variable *M* in time (similarly for *Mf*, *T* and *Tf*).

Equation (1) represents the dynamics of free macrophages ($M$). Every term on the right-hand side of (1) corresponds to a specific aspect in the interaction of the state variables that contributes to the growth or decay of $M$ in time. More specifically, the arrival of $M$ to the site of infection is stimulated by $Mf$(recruitment term); the phagocytosis of $T$ by $M$ can be enhanced by the cell interior released by $Mf$ upon necrosis, where the number of $T$ that can be phagocyted by a single macrophage is controlled by the scaling factor $PTf$ that represents the average bacteria phagocytized by macrophages in vivo. The third term corresponds to the natural death of $M$ and final term accounts for the death of $M$ caused by $T$.

Equation (2) represents the dynamics of macrophages that have phagocytized (*Mf*). The individual terms correspond to $M$ phagocytizing $T$, the natural death of $Mf$, the death of $Mf$ caused by $T$, apoptosis and necrosis, respectively.

Equation (3) represents the dynamics of free *M. tuberculosis* ($T$). The first term corresponds to the logistic growth of $T$. The second term is the release of $T$ into the extracellular medium due to the necrosis of $Mf$, and the last one is $M$ phagocytizing $T$. The number of $T$ released during necrosis is controlled by the scaling factor $PTf$.

Finally, Eq. (4) represents the bacteria that have been phagocytized ($Tf$). The first term corresponds to the logistic growth of $Tf$, with a carrying capacity that is conditioned by the population of $Mf$ since $Tf$ cannot proliferate outside of it. The second term is $M$ engulfing $T$ and the last one is the death of $Tf$ caused by $Mf$.

Notice that in our model the biomass conversion due to phagocytosis follows a kind of mass-energy conservation law typical of Gause-type predator–prey models^70^.

Parameters marked with an $F$ in Eq. (1) (such as ${F}_{\alpha 1}, {F}_{\delta }, etc$) change through the three phases of progression depending on the value of:

$${X}={\int }_{0}^{t}\left(T+Tf\right)dx.$$

This integral can be interpreted as the “history” of bacterial load until time $t$. The transition from phase 1 to phase 2 is specified at a certain instant *critical time 1* in which $X$ attains a given threshold value $X={K}_{m}^{1}$ (Fig. 1B). The new value of such parameter for phase 2 is then given by the formula:

5 $${f}_{1}\left(X\right)=\left({P}_{max}^{1}-{P}_{min}^{1}\right)+{P}_{min}^{1}\left(\frac{{X}^{{n}_{1}\cdot {j}_{1}}}{{X}^{{n}_{1}\cdot {j}_{1}}+{{K}_{m}^{1}}^{{n}_{1}\cdot {j}_{1}}}\right).$$

Similarly, transition from phase 2 to phase 3 occurs at such instant *critical time 2* such that ${X= K}_{m}^{2}$ and the new parameter value for phase 3 is given by

$${f}_{2}\left(X\right)=\left({P}_{max}^{2}-{P}_{min}^{2}\right)+{P}_{min}^{2}\left(\frac{{X}^{{n}_{2}\cdot {j}_{2}}}{{X}^{{n}_{2}\cdot {j}_{2}}+{{K}_{m}^{2}}^{{n}_{2}\cdot {j}_{2}}}\right).$$

Let us explain the ingredients of $${f}_{i}\left(X\right)$$, with $$i=\mathrm{1,2}$$, in more detail. The abrupt and immediate change in parameter values is modelled in a phenomenological way by two Hill functions ($$\frac{{X}^{{n}_{i}\cdot {j}_{i}}}{{X}^{{n}_{i}\cdot {j}_{i}}+{{K}_{m}^{i}}^{{n}_{i}\cdot {j}_{i}}}, i=\mathrm{1,2}$$) that depend on the integral *X*. The constants $${K}_{m}^{i}$$, i = 1,2, represent the threshold values of $$X$$ where the system changes phases. The Hill coefficient, $${n}_{i}$$, i = 1,2, defines the steepness of the Hill function; the higher it is, the more abrupt its change. $${P}_{min}^{i}$$ and $${P}_{max}^{i}$$ are the minimal and maximal parameter values, respectively, between the phase changes. The constant $${j}_{i}$$ is 1 if the Hill function increases and $${j}_{i}=$$ − 1 if it decreases, where $$i=\mathrm{1,2}$$. As shown in Fig. 1C we can see three behaviors: Transient increase or “up-down” ($${j}_{1}$$ = 1, $${j}_{2}$$= − 1), stepwise increase or “up-up” ($${j}_{1}$$ = $${j}_{2}$$ = 1) and stepwise decrease or “down-down” ($${j}_{1}$$ = $${j}_{2}$$ = − 1). Each of the phase-dependent parameters has associated an ($${j}_{1}, {j}_{2})$$ pair as shown in Fig. 1C, as well as maximal and minimal value pairs between phases ($${P}_{min}^{i}, {P}_{max}^{i})$$. To facilitate the implementation of our model, we show in Table 2 the fold-changes in the values of these phase dependent parameters resulting from applying $${f}_{i}\left(X\right)$$.

Model equations can be found on https://github.com/eliezerflores/Tuberculosis-model in MATLAB file Tuberculosis_ODEs.m and in SBML files TBModelPh1.xml, TBModelPh2.xml and TBModelPh3.xml (one file per phase).

### Model simulation

The solution of the model (Eq. 1) was approximated by numerical integration using ode15s function in MATLAB. Initial conditions in Fig. 1D, correspond to the first experimentally determined datapoint, as discussed in detail in^15^. Initial conditions used in Fig. 5B are described in the “Simulation of latent tuberculosis infection” section.

### Identification of bifurcation parameters

To find potential bifurcation parameters, we randomly sampled 500 parameter sets two orders of magnitude around the previously fitted Phase 1 values^15^. We used the Latin Hypercube method in MATLAB to sample from this multidimensional uniform distribution. For each parameter set we computed the number of stable equilibrium points, as described in^15^. We identified those parameters that segregate the monostable from the bistable regimes by systematically performing a two-sample *t*-test using ttest2 function in MATLAB and selected those parameters that rejected the null hypothesis (no difference between mono-and bistable regimes) with a *p* value below or equal to 0.001.

### 1-D bifurcation analysis

For each of the five bifurcation parameters, we varied their values one at the time. For each parametric combination, we obtained all the steady state solutions of Eq. (1) using vpasolve function in MATLAB. We only stored positive real solutions, classifying them as stable or unstable according to the eigenvalues in their Jacobian matrices (all real parts are negative = stable). We explored values of the bifurcation parameters until we found the beginning and the end of the bistable section, i.e., the parameter interval at which the number of stable steady states increases or decreases. The exploration started around the nominal value ${\widehat{P}}_{i}$ of the $i$-th bifurcation parameter by considering the interval [${\widehat{P}}_{i}\cdot 0.1,{\widehat{P}}_{i}\cdot 10]$ with 100 steps, i.e., a step size of $({\widehat{P}}_{i}\cdot 10-{\widehat{P}}_{i}\cdot 0.1)/100$. The last values of ${P}_{i}$ registered before a change of stability was observed are the bifurcations given by the threshold values ${P}_{i}^{-}$ (for a change of mono to bistability) or ${P}_{i}^{-}$ (for a change of bi to mono-stability). We performed this assay three times, one for each phase parameter set.

### 2-D bifurcation analysis

Using the same methodology as for the 1-D bifurcation diagrams we varied two parameters simultaneously and obtained their solutions. Stable solutions were classified as (a) monostable persistent infection, (b) bistable, and (c) monostable bacterial clearance. As for the 1-D diagrams, we explored the interval [${\widehat{P}}_{i}\cdot 0.1,{\widehat{P}}_{i}\cdot 10]$ with 100 steps, i.e., a step size of $({\widehat{P}}_{i}\cdot 10-{\widehat{P}}_{i}\cdot 0.1)/100$ around the nominal values ${\widehat{P}}_{i}$, resulting in 100 × 100 matrices with each cell being a solution classified as (a), (b) or (c). We then visualize the matrix using imagesc on MATLAB. Additionally, for some parameter combinations (like $PTf\times {F}_{{\mathrm{\alpha }}_{1}}$), we changed the value of a third parameter, this single change was maintained while the other two parameters varied around their thresholds. We explored all possible bifurcation parameter combinations and showed only those who were the most interesting. We only performed this assay for parameters in phase 1 state.

### Simulation of latent tuberculosis infection

The mathematical model was able to replicate in vivo data of a LTBI in B6D2F1. In the experimental assay^53^, mice were infected with 4 × 10^3^ H37Rv bacteria. For our numerical integrations we considered an initial condition of 1 × 10^3^ for free bacteria ($T$) because we assumed that not all the inoculated bacteria reach the infection site. Macrophage ($M$) initial condition was 173,654.91, a number obtained by averaging macrophage counts on homeostasis^71^. $Mf$ and $Tf$ initial values were zero. As for the parameters, we started selecting a random parameter set (previously generated by a Latin hypercube sampling from the nominal parameter set as explained in^15^) that had a critical time 1 near 90. We then varied ${F}_{{\upbeta }_{5}}$, ${F}_{\delta }$ and ${F}_{{\mathrm{\alpha }}_{1}}$ heuristically until we obtained the desired outcome (Table 1).

### Supplementary Information

Supplementary Information.

## Data Availability

The code is stored in our GitHub folder https://github.com/eliezerflores/Tuberculosis-model/ and will be made publicly available upon acceptance.
