# Publisher Correction: Bifurcation analysis of a tuberculosis progression model for drug target identification

**DOI:** 10.1038/s41598-023-46856-9

**Published:** 2023-11-17

**Authors:** Eliezer Flores-Garza, Rogelio Hernández-Pando, Ibrahim García-Zárate, Pablo Aguirre, Elisa Domínguez-Hüttinger

**Affiliations:** 1 Departamento de Biología Molecular y Biotecnología, Instituto de Investigaciones Biomédicas, Universidad Nacional Autónoma de México, Ciudad Universitaria, 04510 Mexico, Mexico; 2 Sección de Patología Experimental, Departamento de Patología, Instituto Nacional de Ciencias Médicas y Nutrición Salvador Zubirán, Vasco de Quiroga 15, Belisario Domínguez Secc. 16, Tlalpan, 14080 Mexico City, Mexico; 3 Facultad de Ciencias, Universidad Nacional Autónoma de México, Ciudad Universitaria, Coyoacán, 04510 Mexico City, Mexico; 4 Departamento de Matemática, Universidad Técnica Federico Santa María, Casilla 110-V, Valparaíso, Chile

Correction to: *Scientific Reports* 10.1038/s41598-023-44569-7, published online 16 October 2023

The original version of this Article contained an error in Figure 3, where the labels on panels A, B and C were partly omitted. The original Figure 3 and accompanying legend appear below.

**Figure 3:**
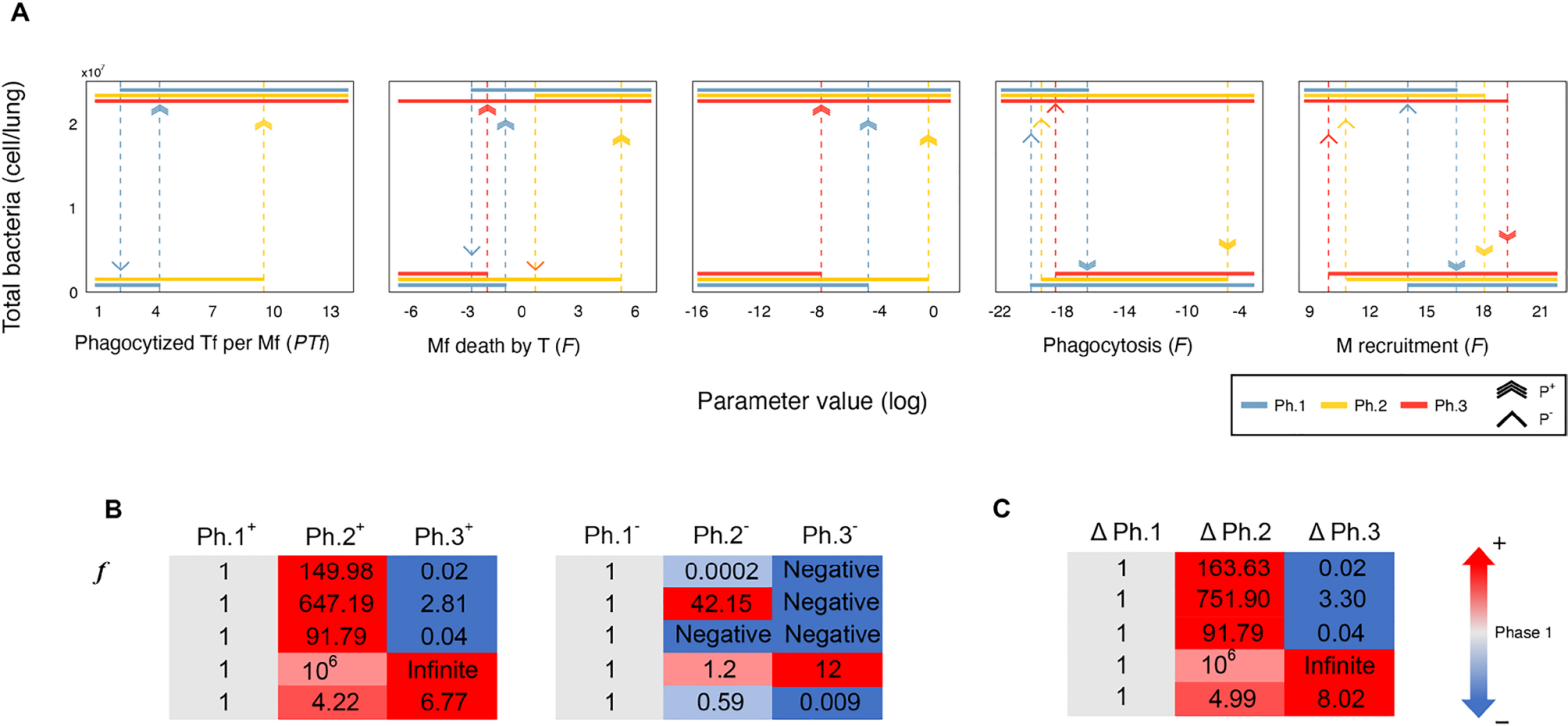
Disease progression affects the sensitivity of the clinical phenotype to changes in the bifurcation parameters. (**A**) Bifurcation diagrams for the five bifurcation parameters across the 3 phases. Unstable solutions are not shown for clarity. Dotted vertical arrows denote threshold parameter values when an abrupt change in stability happens, from bacterial clearance to invasive infection or vice versa. (**B**) Right (*P*^−^) and left (*P*^+^) threshold parameter values enclosing the bistable region change as the disease progresses. Each row corresponds to one of the 5 bifurcation parameters, and the columns are the *P*^−^ (left panel) *P*^+^ (right panel) threshold parameter values for each phase, normalized to its phase 1—specific threshold value. (**C**) Size of the bistable region. For negative *P*^−^, Δ*P* corresponds to *P*^+^.

The original Article has been corrected.

