# Supplementary material for: Bifurcation analysis of a tuberculosis progression model for drug target identification
Source: Sci Rep. 2023 Oct 16;13:17567. doi: 10.1038/s41598-023-44569-7 (PMC10579266; doi:10.1038/s41598-023-44569-7)
Supplement: Supplementary file 1 — Supplementary Information. [file 41598_2023_44569_MOESM1_ESM.pdf]

Supplementary information: Bifurcation analysis of a tuberculosis progression model for drug target identification

Eliezer Flores-Garza, Rogelio Hernández-Pando, Ibrahim García-Zárate, Pablo Aguirre, Elisa Domínguez-Hüttinger*

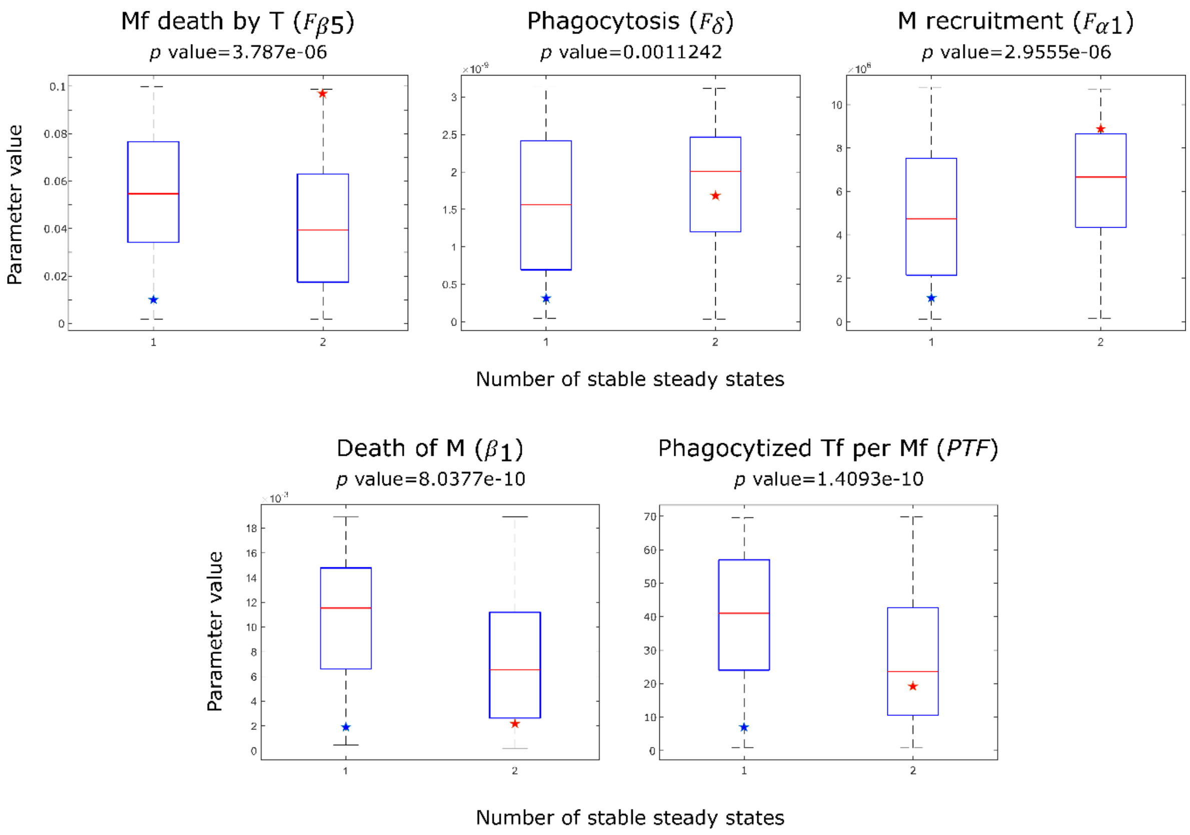

Figure S1. Identification of bifurcation parameters. The distribution of values of five of the 15 parameters that show a significant difference respect to monostable and bistable regimes. Blue stars are the value of the parameter in the nominal parameter set and red stars the value in the bistability set, red lines represent the median of the distribution of parameter values. *P* value is reported on the top of each box plot.

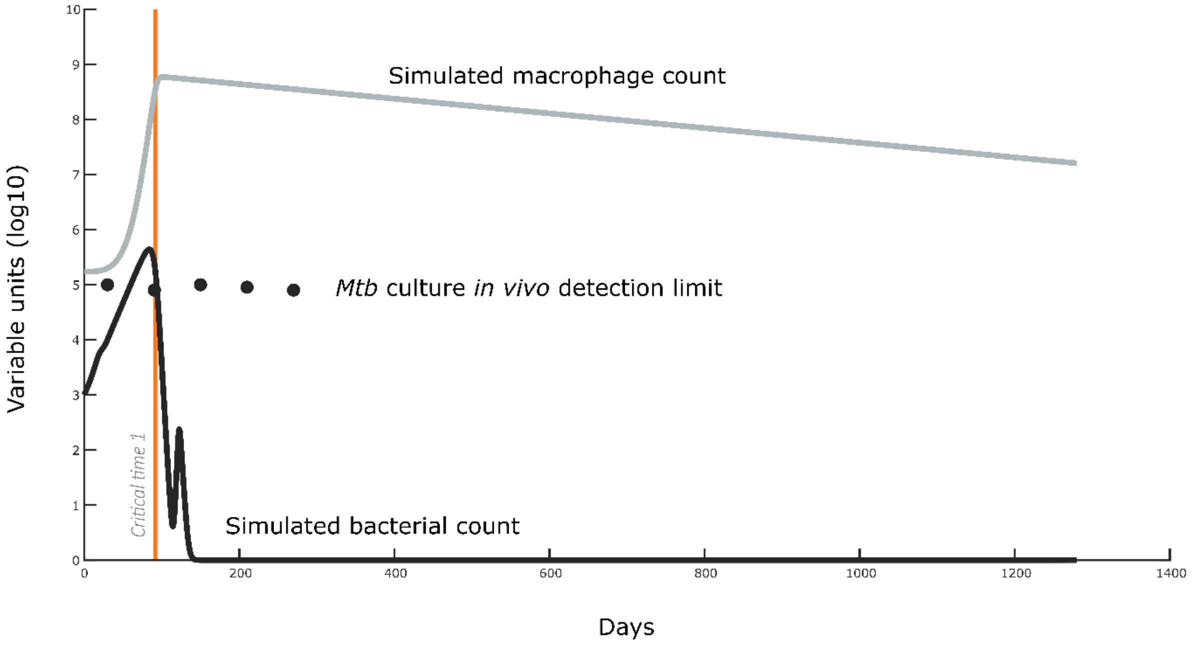

Figure S2. Numerical integration of the model with an integration time equal than the highest lifespan expectancy of lab mice [1]. Bacteria load counts (CFU) from LTBI mice lung (dots) [2] vs total bacteria (black line) of the model, gray line represents total macrophages of the model. Orange vertical line is model and *in vivo* data critical time 1.

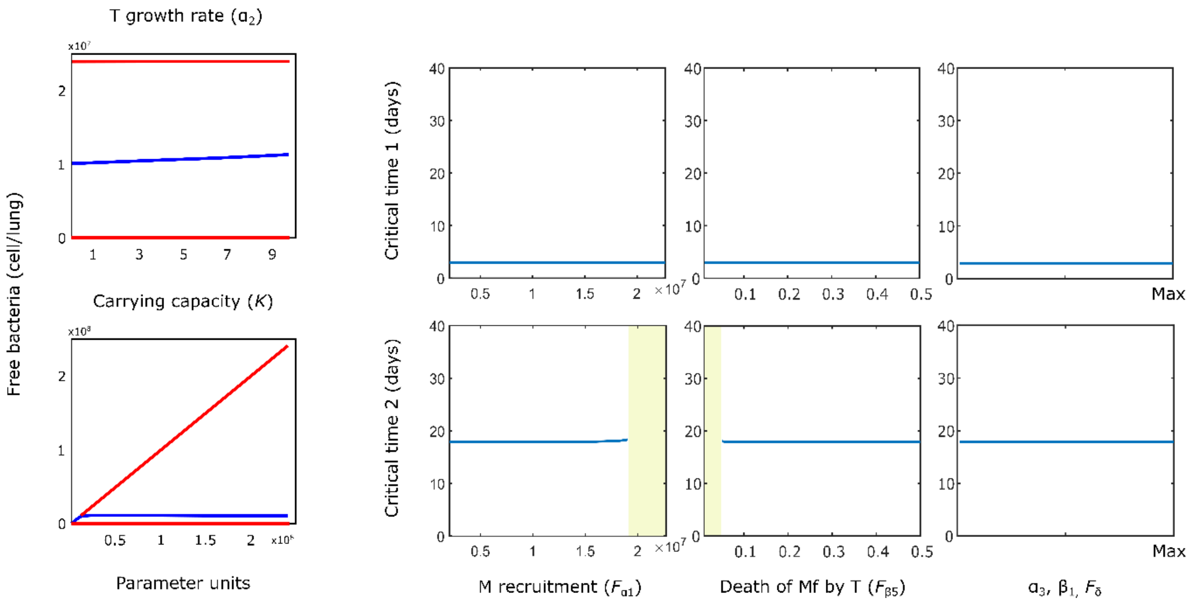

Figure S3. Left: 1-D Bifurcation analysis of α_2 and K, colors and axes are coded as in figure 2. Right: Critical times 1 and 2 (Y axis) in response to single parameter variation (X axis). The last graphic groups multiple parameters that showed the same response when modulated.

Supplementary Table 1: Total bacteria counts from figure 1c

Dataset (Ref)	Bacterial challenge	Measured bacteria (10^6 CFU)	Measurement times (Days)
[3]	$2.5x10^5$	[0.3, 0.5, 1.6, 14, 19, 16, 24]	[1, 3, 7, 14, 21, 28, 60]
[4]	$2.5x10^5$	[0.6, 1, 4, 13, 13, 29, 38]	[1, 3, 7, 14, 21, 28, 60]
[5]	$2.5x10^5$	[0.2, 8, 28, 29]	[3, 14, 21, 28]
[6]	$1x10^6$	[0.3, 0.4, 2, 32, 27, 49, 68]	[1, 3, 7, 14, 21, 28, 60]

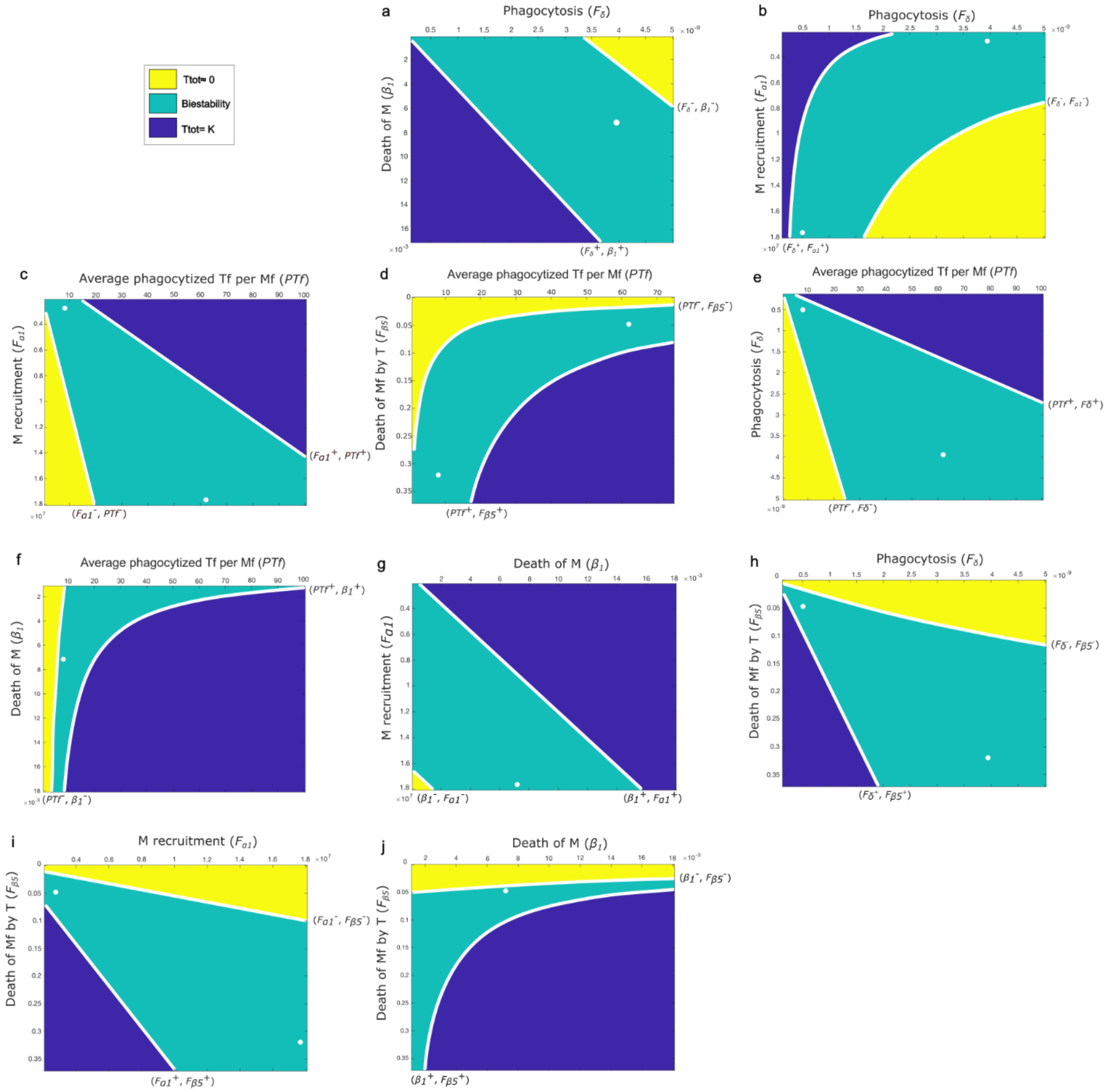

Figure S4. Two-dimensional bifurcation analysis of all possible combinations of the 5 bifurcation parameters. The nominal value of each phase 1 parameter is shown as red lines.

Supplementary references

[1] P. Sengupta, 'The Laboratory Rat: Relating Its Age With Human's', *Int J Prev Med*, vol. 4, no. 6, p. 624, Jun. 2013, Accessed: Oct. 05, 2022. [Online]. Available: /pmc/articles/PMC3733029/

[2] A. K. Arriaga, E. H. Orozco, L. D. Aguilar, G. A. W. Rook, and R. Hernández Pando, 'Immunological and pathological comparative analysis between experimental latent tuberculous infection and progressive pulmonary tuberculosis', *Clin Exp Immunol*, vol. 128, no. 2, pp. 229–237, 2002, doi: 10.1046/j.1365-2249.2002.01832.x.

[3] E. I. Bini *et al.*, 'The influence of sex steroid hormones in the immunopathology of experimental pulmonary tuberculosis', *PLoS One*, vol. 9, no. 4, pp. 2–10, 2014, doi: 10.1371/journal.pone.0093831.

[4] A. Montoya-Rosales *et al.*, 'lysX gene is differentially expressed among Mycobacterium tuberculosis strains with different levels of virulence', *Tuberculosis*, vol. 106, pp. 106–117, 2017, doi: 10.1016/j.tube.2017.07.005.

[5] G. J. Baay-Guzman *et al.*, 'Dual role of hypoxia-inducible factor 1 α in experimental pulmonary tuberculosis: Its implication as a new therapeutic target', *Future Microbiol*, vol. 13, no. 7, pp. 785–798, 2018, doi: 10.2217/fmb-2017-0168.

[6] R. Hernández-Pando, D. Aguilar, M. L. G. Hernández, H. Orozco, and G. A. W. Rook, 'Pulmonary tuberculosis in BALB/c mice with non-functional IL-4 genes: Changes in the inflammatory effects of TNF-α and in the regulation of fibrosis', *Eur J Immunol*, vol. 34, no. 1, pp. 174–183, 2004, doi: 10.1002/eji.200324253.
